# Laparoscopic gastric partitioning gastrojejunostomy for an unresectable duodenal malignant tumor

**DOI:** 10.4103/0972-9941.18997

**Published:** 2005-09

**Authors:** Toshifumi Matsumoto, Koichi Izumi, Akio Shiromizu, Kohei Shibata, Masayuki Ohta, Seigo Kitano

**Affiliations:** Department of Surgery I, Oita University, Faculty of Medicine, Oita 879-5593, Japan

**Keywords:** bypass, gastrojejunostomy, laparoscopic, palliative, unresectable

## Abstract

As a palliative bypass for unresectable gastric or periampullary cancer, gastrojejunostomy (GJ) is sometimes associated with postoperative delayed gastric emptying. We report the successful laparoscopic application of this procedure in a 78-year-old man with duodenal obstruction. Computed tomography revealed a mass in the duodenum along with multiple masses in the liver. A radiological image showed an ulcerative tumour in the third portion of the duodenum occluding the lumen. He was diagnosed as having an unresectable duodenal cancer with multiple liver metastases. He needed palliative bypass surgery. Laparoscopically, the stomach was partially divided using an endoscopic autosuture device, and end-to-side GJ was performed successfully. He was given a normal diet on the fourth postoperative day, and there was no delayed gastric emptying. Laparoscopic gastric partitioning GJ is a feasible and safe procedure to prevent postoperative delayed gastric emptying in case of malignant duodenal obstruction.

Many patients with gastric or periampullary cancer are diagnosed as having cancer at an advanced stage, in which the prognosis is quite poor. Patients with advanced gastric cancer often have gastric outlet obstruction. Patients with unresectable periampullary cancer also tend to have locally advanced tumours involving neighbouring organs such as the duodenum or the transverse colon. These patients often suffer from repeated vomiting. To preserve the quality-of-life for patients with unresectable gastric or periampullary cancer, a gastrojejunostomy (GJ) is often performed. However, some patients who have undergone GJ are not satisfied because of delayed gastric emptying or bleeding from the tumour.[1] Gastric partitioning GJ has been performed to avoid delayed gastric emptying.[2] The stomach is partially partitioned between the proximal and distal parts; the proximal part of the stomach is anastomosed to the proximal part of the jejunum [Figure 1]. We report a patient who underwent laparoscopic gastric partitioning GJ for an unresectable duodenal cancer.

**Figure 1:**
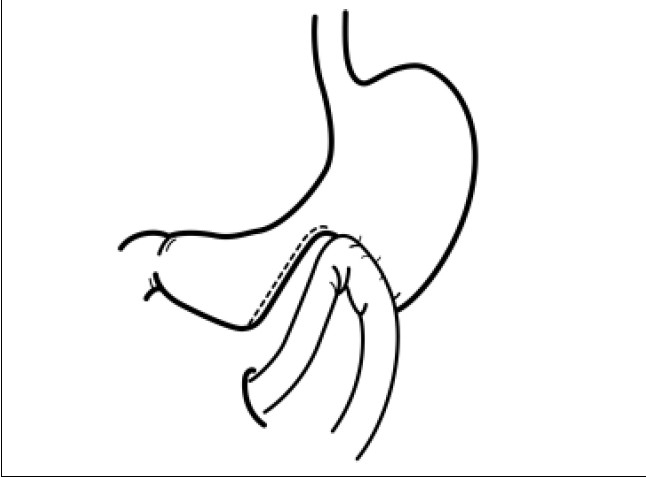
Schema of gastric partitioning gastrojejunostomy

## CASE REPORT

**Figure 2:**
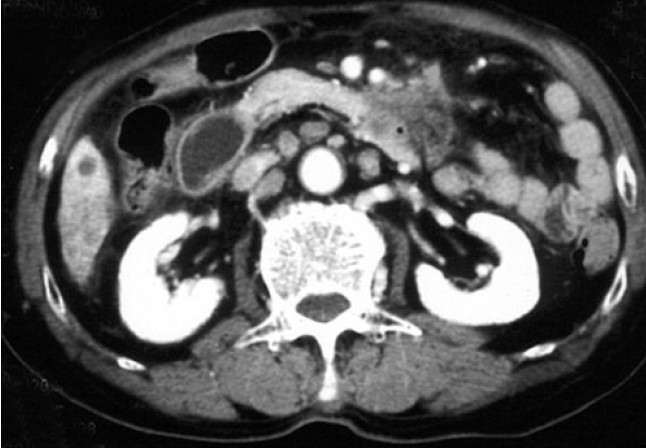
Computed tomography reveals a tumor in the third portion of the duodenum.

**Figure 3:**
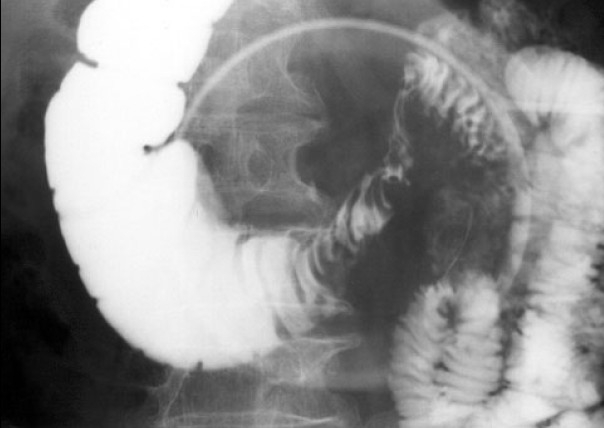
Upper gastrointestinal image showed the ulcerative tumor in the third portion of the duodenum narrowing the lumen.

**Figure 4:**
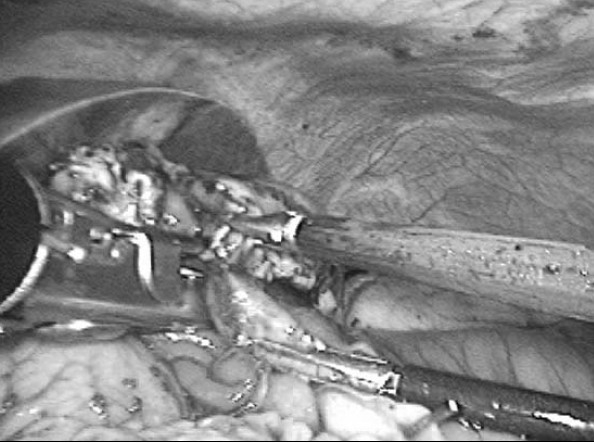
End-to-side gastrojejunostomy was created using an endoscopic autosuturing stapler.

**Figure 5:**
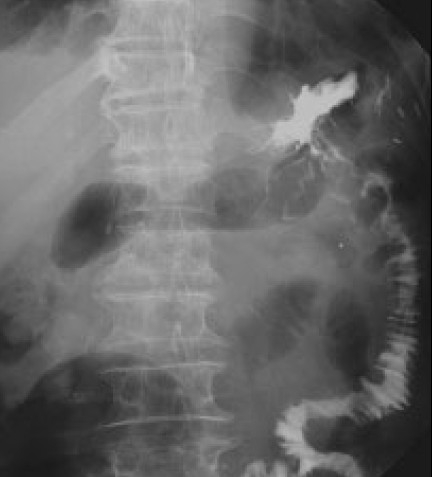
Upper gastrointestinal images shows that most barium medium passes the anastomotic portion, While a little amount passes through the tunnel of the lessere curvature

A 78-year-old man was referred to our hospital because of repeated vomiting for 4 days. Computed tomography revealed a duodenal tumour [Figure 2] along with multiple liver masses. A radiological image with barium contrast medium showed a severe stenosis in the third portion of the duodenum [Figure 3]. The patient was diagnosed as having duodenal cancer with multiple liver metastases. Curative resection was not indicated, and laparoscopic gastric partitioning GJ as a palliative surgery was applied. We performed this procedure with the patient in the supine position, the standard position for laparoscopic gastric surgery. Five ports were inserted and a pneumoperitoneum was created. The great omentum was dissected using an ultrasonic activated scalpel (SonoSurg, Olympus Corp. Co., Tokyo, Japan) along the great curvature. The lower part of the stomach was divided using a 60-mm EndoGIA II stapler (USSC, Norwalk, CT, USA) to maintain a tunnel of approximately 3 cm in diameter in the lesser curvature of the stomach. A loop of jejunum, located 25 cm distal from the ligament of Treitz, was brought up in an antecolic fashion. The jejunum was connected to the proximal part of the divided stomach. After the site was chosen for the anastomosis between the stomach and the jejunum, a small jejunotomy was made on the opposite side of the mesenterium, as was a small gastrotomy on the posterior side of the stomach. For the anastomosis, the 60-mm EndoGIA II stapler was used [Figure 4]. After firing and removing the staples, the inverted portions of the linear staple line were inspected for luminal patency and haemostasis. The incisional sites of the stomach and the jejunum were closed by manual suturing. Upon completion of the anastomosis, a gastrofiberscopic examination was performed to check for leakage and stenosis of the anastomosis. The operative time was 245 min, and the intraoperative estimated blood loss was 20 ml. The nasogastric tube was removed the next morning. The patient was given a normal diet on the fourth postoperative day. Radiographic examination showed a good patency through the GJ on the eighth postoperative day [Figure 5]. We planned to discharge the patient, but a cerebral infarction in the posterior lobe occurred on the 12th postoperative day. Delayed gastric emptying did not occur during the patient's stay at the hospital for the care of his infarction. The patient was discharged on the 30th postoperative day.

## DISCUSSION

Pyloric stenosis due to advanced gastric cancer or duodenal obstruction due to periampullary cancer diminishes the patient's quality-of-life. Patients with limited life expectancy suffer more if they cannot eat sufficiently. Bypass surgery has been considered one of the better choices for the relief of such suffering. However, conventional GJ is often associated with such problems as delayed gastric emptying and bleeding from the tumour surface.[3] In unresectable gastric cancer, tumour growth sometimes results in early obstruction of the anastomotic lume. Palliative surgery should resolve these problems effectively.

Devine introduced a type of bypass procedure consisting of transection of the stomach and anastomosis between the jejunal loop and the proximal stump of the divided stomach.[4] This procedure has often been used in patients with unresectable gastric or pancreatic cancer.[2] Transection of the stomach may prevent postoperative delayed gastric emptying and haemorrhage from the tumour caused by contact with food. However, it was reported that gastric secretion and bleeding from the tumour could lead to a rupture of the transected distal part of the stomach.[5] Schantz et al[6] reported improved quality of life through the use of a modified Devine procedure with gastric partitioning, in which manipulation is simplified by the use of a linear cutter. This method minimizes contact between food and the tumour and allows endoscopic examination through the tunnel in the lesser curvature of the stomach. Kaminishi et al[2] also reported that gastric partitioning GJ improved both the quality-of-life and the prognosis for patients with unresectable gastric cancer.

Among patients with periampullary cancer, 10–20% of patients develop gastroduodenal obstruction after a biliary-digestive bypass alone.[7] Prophylactic GJ to prevent duodenal obstruction during exploratory laparotomy was recommended in patients with unresectable periampullary carcinoma. But delayed gastric emptying and gastrointestinal bleeding have been reported in patients who underwent prophylactic GJ. In a retrospective study by the Memorial Sloan-Kettering Cancer Center,[8] the perioperative morbidity rate after prophylactic GJ increased significantly without improving prognosis. Therefore, a safe and effective GJ has been needed.

Choi[9] reported that patients treated with laparoscopic GJ did not experience complications in the early postoperative period. Compared with open GJ, laparoscopic GJ reduced morbidity, mortality and the incidence of postoperative complications, allowed for earlier oral feeding, and shortened the hospital stay.[10] In addition, laparoscopic exploration and staging for gastric cancer or pancreatic cancer are successfully performed without unnecessary laparotomy. When a tumour is unresectable, palliative laparoscopic GJ can be performed during laparoscopy and will improve the patient's quality of last life.

In conclusion, we report the first case of laparoscopic gastric partitioning GJ. It is technically feasible and safe, and frees the patient from symptoms of duodenal obstruction. This procedure seems to be effective for preventing delayed gastric emptying.
